# The Road to Sepsis in Geriatric Polytrauma Patients—Can We Forecast Sepsis in Trauma Patients?

**DOI:** 10.3390/jcm13061570

**Published:** 2024-03-09

**Authors:** Cédric Niggli, Philipp Vetter, Jan Hambrecht, Hans-Christoph Pape, Ladislav Mica

**Affiliations:** Department of Trauma Surgery, University Hospital Zurich, 8091 Zurich, Switzerland

**Keywords:** sepsis, polytrauma, geriatric polytrauma

## Abstract

**Background:** Sepsis is a leading cause of mortality in polytrauma patients, especially beyond the first week, and its management is vital for reducing multiorgan failure and improving survival rates. This is particularly critical in geriatric polytrauma patients due to factors such as age-related physiological alterations and weakened immune systems. This study aimed to investigate various clinical and laboratory parameters associated with sepsis in polytrauma patients aged < 65 years and ≥65 years, with the secondary objective of comparing sources of infection in these patient groups. **Methods:** A retrospective cohort study was conducted at the University Hospital Zurich from August 1996 to December 2012. Participants included trauma patients aged ≥16 years with an Injury Severity Score (ISS) ≥ 16 who were diagnosed with sepsis within 31 days of admission. Patients in the age groups < 65 and ≥65 years were compared in terms of sepsis development. The parameters examined included patient and clinical data as well as laboratory values. The statistical methods encompassed group comparisons with Welch’s *t*-test and logistic regression. **Results:** A total of 3059 polytrauma patients were included in the final study. The median age in the group < 65 years was 37 years, with a median ISS of 28. In the patient group ≥ 65 years, the median age was 75 years, with a median ISS of 27. Blunt trauma mechanism, ISS, leucocytosis at admission, and anaemia at admission were associated with sepsis in younger patients but not in geriatric patients, whereas sex, pH at admission, lactate at admission, and Quick values at admission were not significantly linked with sepsis in either age group. Pneumonia was the most common cause of sepsis in both age groups. **Conclusions:** Various parameters linked to sepsis in younger polytrauma patients do not necessarily correlate with sepsis in geriatric individuals with polytrauma. Hence, it becomes critical to recognize imminent danger, particularly in geriatric patients. In this context, the principle of “HIT HARD and HIT EARLY” is highly important as a proactive approach to effectively address sepsis in the geriatric trauma population, including the preclinical setting.

## 1. Background

The leading cause of death beyond the first week in polytrauma patients is septic complications [1]. This susceptibility to infection arises from Compensatory Anti-Inflammatory Response Syndrome (CARS), a state of immunological fatigue following trauma [2]. Uprising sepsis weakens patient physiology and destroys organs as well as the central and peripheral nervous systems. The treatment principle “HIT HARD and HIT EARLY” has demonstrated its capacity to reduce the occurrence of multiorgan failure (MOF), ultimately resulting in enhanced survival [3].

While sepsis affects individuals of all ages, it poses a particular challenge for geriatric polytrauma patients compared to younger populations. This distinction arises from the complex interplay of factors inherent to older adults, including age-related physiological changes, comorbidities, and a potentially compromised immune system. As the global demographic landscape continues to shift toward an aging society, understanding and addressing the distinctive challenges posed by sepsis in geriatric polytraumas is becoming increasingly crucial.

To address this need, this study group collaborated with IBM to develop the IBM WATSON Trauma Pathway Explorer©, a visual analytics tool that predicts outcomes in severely injured individuals [4,5,6,7,8]. This validated and interactive tool utilizes clinical and laboratory values to predict various outcomes, including the occurrence of sepsis after a patient’s admission. Notably, users can analyze the risk of sepsis in polytrauma patients across different age groups.

The comparison of different parameters associated with sepsis in different age groups is essential due to the distinct physiological and immunological differences between these age groups:

- Younger patients might show stronger links to traditional markers like leucocytes and injury severity.
- Geriatric patients might have different risk factors due to pre-existing conditions or immune response variations.

The primary objective of this study was to examine different clinical and laboratory parameters upon admission of polytrauma patients aged < 65 and ≥65 years regarding the development of sepsis. The secondary aim was to compare the sources of infection in polytrauma patients with sepsis aged < 65 and ≥65 years.

## 2. Methods

### 2.1. Study Design

This article’s research adhered to the STROBE Statement, a guideline for observational studies in epidemiology [9]. The data used in this study were acquired through a retrospective cohort study conducted at the University Hospital Zurich, covering the period from August 1996 to December 2012.

### 2.2. Participants

The inclusion criteria for patients were age ≥ 16 years and an Injury Severity Score (ISS) ≥ 16. As widely acknowledged, polytrauma is defined by an ISS ≥ 16 points, indicating a condition where a person sustains multiple traumatic injuries to different body regions simultaneously [10]. The study focused on patients who were directly admitted to the trauma bay. Patients with missing data on the presence or absence of sepsis were excluded from the study.

### 2.3. Outcomes

The outcome investigated was the occurrence of sepsis within 31 days after the patient’s admission to the trauma hospital. The development of sepsis was studied separately for the age groups < 65 and ≥65 years.

To classify as sepsis, a Systemic Inflammatory Response Syndrome (SIRS) score of ≥2 was required, in conjunction with evidence of an additional infectious source [11]. The confirmation of infection was established through clinical sepsis criteria, which encompass signs of organ dysfunction, hypotension, and hypoperfusion, or through microbiological detection [11]. Sepsis had to manifest at any point during the 31-day observation period. SIRS was assessed based on the presence of two or more of the following criteria: body temperature > 38 °C or <36 °C, heart rate > 90 bpm, respiratory rate > 20 breaths/min, and white blood cell count > 12,000/µL or <4000/µL [11]. SIRS was evaluated over the initial 31 days following admission.

Notably, this study adhered to the traditional sepsis definition outlined by the ACCP/SCCM Consensus Conference Committee [11]. Although an alternative definition for sepsis, known as the Sepsis-3 criteria [12], has emerged in recent years, the data collected from this hospital cohort employed established and widely recognized older criteria. Furthermore, recent research has suggested that the older definition of sepsis may outperform the newer definition [13].

### 2.4. Parameters

The age groups < 65 and ≥65 years were examined for the development of sepsis. The term “geriatric polytrauma” typically refers to the occurrence of multiple traumatic injuries in an older adult. Although there is no universally agreed-upon age at which a person is classified as “geriatric” in the context of trauma, it is generally associated with individuals who are 65 years of age or older. This age cutoff is commonly used in the medical literature and in clinical practice [14].

The analysis incorporated fundamental parameters, encompassing patient information, clinical data, and laboratory results at admission, to explore the correlation between these parameters and the onset of sepsis in both age groups.

Patient data consisted of age and sex. Clinical parameters related to trauma assessment included the mechanism of trauma (blunt or penetrating), ISS, temperature at admission, Glasgow Coma Scale (GCS) at the accident site, and ATLS shock classification. The Abbreviated Injury Scales (AIS) for the different body regions were also included. The scale ranges from 1 to 6 according to the severity of the injury (minor, moderate, serious, severe, critical, maximum). The laboratory data comprised leucocytes, C-reactive protein (CRP), pH, lactate, hemoglobin, hematocrit, and Quick values at admission. All the parameters were subjected to analysis for both age groups. The following sources of infections were documented for all patients: pneumonia, bacteremia, catheter-related bloodstream infections (CRBSIs), wound infections, urinary tract infections (UTIs), central nervous system (CNS) infections, intraabdominal infections, osteomyelitis, and other infections.

### 2.5. Data Measurement

Age, sex, trauma mechanism, ISS, and temperature at admission [°C] were extracted from the emergency room admission records. GCS at the accident site was obtained from the rescue service protocol. Point-of-Care-Testing (POCT) performed in the emergency room provided data on pH, lactate [mmol/L], hemoglobin [g/dL], hematocrit [%], and Quick scores [%]. Leucocytes [WBC/µL] and CRP [mg/L] levels were determined in the Department of Clinical Chemistry at the University Hospital Zurich.

### 2.6. Statistics

The baseline characteristics of the patient sample (overall, <65 years, ≥65 years) were summarized using medians along with interquartile ranges (IQRs) for interval, ratio, and ordinal data, while percentages were used for binary variables. Differences among these groups were evaluated using the Wilcoxon rank sum test for numerical data and Pearson’s Chi-squared test for categorical variables, with *p* < 0.05 indicating statistical significance.

To assess the central tendency of different parameters in the sepsis and non-sepsis cohorts within the age groups <65 years and ≥65 years, Welch’s *t*-tests were employed [15,16].

Binary logistic regression analysis was also conducted to investigate the influence of patient characteristics and laboratory parameters on sepsis development in both age groups. Odds ratios (ORs) with corresponding 95% confidence intervals (CIs) were computed [17].

No imputation method was utilized for missing values. Statistics were performed with R-4.2.2 (https://www.r-project.org/, accessed on 1 December 2023).

### 2.7. Ethics

The study adhered to the guidelines for good clinical practice and the Helsinki guidelines. The analysis of trauma patient records was approved by the University Hospital Zurich’s ethics commission and the Zurich government upon the development of the database (Nr. StV: 1-2008). They again re-approved it for the development of the WATSON Trauma Pathway Explorer© (BASEC: 2021-00391).

## 3. Results

### 3.1. Patient Selection

**Figure 1:**
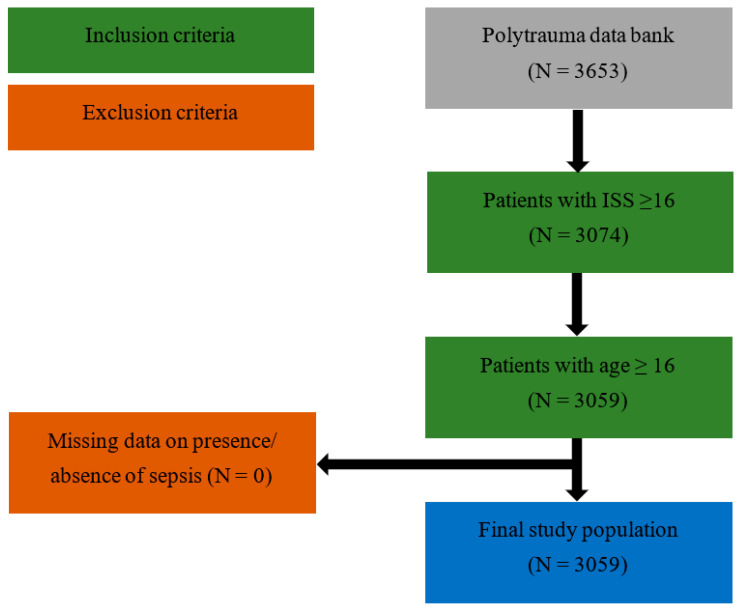
Flowchart of patient selection.

The polytrauma database consisted of 3653 patients treated between 1996 and 2012. The data preparation yielded 3074 patients (84.2%) with an ISS ≥ 16 and 3059 patients (83.7%) aged ≥ 16 years. No patient was excluded due to missing data on the presence or absence of sepsis (0%), resulting in a final study population of 3059 patients (83.7%). The number of participants at each stage is shown in Figure 1.

### 3.2. Descriptive Data

In total, 3059 patients were included. Among the patients aged < 65 years, most participants were men, while this proportion decreased significantly among those aged ≥65 years (77% vs. 60%, *p* < 0.001). The group < 65 years had a median age of 37 years and a median ISS of 28. In the patient group ≥ 65 years, the median age was 75 years, with a median ISS of 27. Young patients suffered significantly more penetrating traumas than patients aged ≥65 years (9% vs. 3%, *p* < 0.001). Older patients experienced more severe head injuries, whereas younger patients had more severe injuries to the thorax, abdomen, and extremities. In the group <65 years, patients spent significantly more time in the ICU or hospital than older patients ≥65 years did (5 days/15 days vs. 2 days/7 days, *p* < 0.001). However, older patients experienced significantly more deaths during hospitalization and within 72 h since admission compared to younger patients (50%/37% vs. 25%/18%, *p* < 0.001). Young patients developed sepsis significantly more often than older patients did (18% vs. 12%, *p* < 0.001). ATLS shock class I was more represented in older patients, while shock classes II-IV were more common in the young patient group. The GCS and temperature did not significantly differ. Among the laboratory parameters at admission, the following values differed significantly between the age groups <65 years and ≥65 years: leucocytes (12.5 vs. 11.5 WBC/µL, *p* < 0.001), CRP (3 (1, 4) vs. 3 (1, 7) mg/L, *p* < 0.001), pH (7.33 vs. 7.35, *p* = 0.011), lactate (2.38 vs. 2.00 mmol/L, *p* < 0.001), hemoglobin (11.70 vs. 11.40 g/dL, *p* = 0.018), and Quick (84% vs. 78%, *p* < 0.001). Patients aged <65 years underwent Damage Control Surgery (DCS) more often than older patients did (53% vs. 39%, *p* < 0.001). In contrast, patients ≥65 years more often did not undergo any intervention (30% vs. 13%, *p* < 0.001). The total values for each variable were also included (Table 1).

### 3.3. Main Results

#### 3.3.1. Central Tendency of Different Parameters with Sepsis in the Two Age Groups

Table 2 illustrates how different clinical parameters are distributed by sepsis in the groups < 65 years and ≥65 years. Given are means, Welch’s *t*-tests, and *p*-values.

Patients with sepsis patients aged <65 years had significantly greater ISSs (*p* < 0.001). A greater mean temperature was observed in patients with septic polytrauma ≥65 years (*p* = 0.01). Patients who developed sepsis had higher leucocyte counts and CRP levels at admission in both age groups, whereas the difference was significant only for patients < 65 years (*p* = 0.04 and 0.04, respectively). The pH and lactate levels at admission did not significantly differ in both age groups. Significant lower hemoglobin and hematocrit levels at admission were observed in patients with sepsis <65 years (*p* = 0.02 and 0.01). The Quick values at admission demonstrated no difference in patients who developed sepsis in both age groups (Table 2).

#### 3.3.2. Correlations of Different Parameters with Sepsis in the Two Age Groups

In the patient group <65 years, univariate logistic regression analysis revealed significant associations between sepsis and blunt trauma (OR = 1.69, *p* = 0.013), ISS (OR = 1.02, *p* < 0.001), ATLS shock class (OR = 1.15, *p* = 0.016), leucocytes (OR = 1.02, *p* = 0.027), CRP (OR = 1.00, *p* = 0.010), hemoglobin (OR = 0.96, *p* = 0.027) and hematocrit at admission (OR = 0.98, *p* = 0.012). In the patient group ≥65 years, the GCS at the site (OR = 1.07, *p* = 0.019) and CRP level at admission (OR = 1.01, *p* = 0.007) were associated with sepsis according to univariate logistic regression analysis, as detailed in Table 3.

Subsequently, multivariate logistic analysis incorporated all the significant univariate variables (except for the AIS). The analysis confirmed that only blunt trauma (OR = 1.85, *p* = 0.034), ISS (OR = 1.02, *p* < 0.001), leucocytes (OR = 1.04, *p* < 0.001) and CRP at admission (OR = 1.00, *p* = 0.048) were found to be independent risk factors for sepsis in the age group < 65 years, whereas GCS at site (OR = 1.11, *p* = 0.006) and CRP at admission (OR = 1.01, *p* < 0.001) were found to be independent risk factors for sepsis in the age group ≥ 65 years, as presented in Table 4.

#### 3.3.3. Source of Infection in Polytrauma Patients with Sepsis

The most common source of infection in younger polytrauma patients with sepsis was pneumonia (68%), followed by bacteremia (36%), CRBSI (26%), wound infection (20%) and UTI (15%). Pneumonia (75%) was also the most common cause of sepsis in geriatric patients, followed by bacteremia (37%), wound infections (24%), UTIs (20%), and CRBSIs (19%). Only CNS infections were significantly different between the young and older age groups (9.8% vs. 2.7%, *p* = 0.044). The results are presented in Table 5.

Of the 430 patients <65 years who developed sepsis, 7.4% died from sepsis (n = 32). The proportion of documented deaths from sepsis in patients ≥65 years was greater at 25% (n = 19).

## 4. Discussion

### 4.1. Key Results

Blunt trauma mechanism, ISS, high inflammation values at admission, and anemia at admission were identified as key factors for sepsis only in younger polytrauma patients but not in geriatric patients. Higher GCS and CRP levels were associated with sepsis in the geriatric polytrauma group. Gender, pH at admission, lactate at admission, and Quick at admission had little impact on the development of sepsis in either age group.

In both younger and geriatric polytrauma patients with sepsis, pneumonia was the most common source of infection, accounting for 68% and 75%, respectively. One-quarter of all geriatric polytrauma patients with sepsis died as a result of sepsis.

### 4.2. Limitations

This study has several limitations. First, it did not account for the temporal progression of laboratory parameters or make any effort to include additional factors such as interleukin-6 or procalcitonin. Second, due to the lack of fully documented patient comorbidities, the patients’ underlying health conditions were not always considered. Third, it was not possible to determine the complete number of patients who required ventilation and intubation upon hospital admission due to missing data, which may affect the development of respiratory infections that could lead to sepsis. Fourth, the database was initiated on 1 August 1996, with continuous data collection, and it is reasonable to assume that there may have been fluctuations over the years in terms of admitting polytrauma patients, leading to potential inconsistencies in patient inclusion in the registry. Fifth, variations in measurement methods for different laboratory values throughout the collection of the data may have resulted in minor discrepancies in blood levels. Sixth, there were some missing data upon admission (particularly for temperature, CRP, pH, and Quick), which may be acceptable given the extensive patient sample. Furthermore, the study does not provide any insights into the long-term survival rate of the patient cohort. Finally, minor changes in treatment protocols or hygiene policies over the past decade may have had an impact on the occurrence of sepsis following surgical procedures.

### 4.3. Interpretation

Polytrauma patients constitute a diverse group of patients, including those aged <65 years and ≥65 years. Age-related differences in immune function, comorbidities, and physiological responses to trauma may influence susceptibility to sepsis in these two age groups.

In this study, a significantly lower incidence of sepsis was observed in the group ≥65 years than in patients aged <65 years. This observation can be partially attributed to the significantly greater occurrence of early death within 72 h among patients ≥65 years of age, which frequently precludes the timely diagnosis of sepsis. In a single trauma center analysis, Kocuvan et al. revealed a significant increase in the occurrence of sepsis among elderly individuals aged ≥65 years who had sustained major trauma [18]. Several studies identified older age as an independent risk factor for sepsis following trauma [19,20,21,22,23]. However, in a study by van Wessem et al., severely injured patients ≥70 years did not develop more infectious complications (such as sepsis) than younger patients did [24]. Similarly, Tong et al. and Chung et al. demonstrated that age is not a significant risk factor for sepsis in patients with multiple traumas [25,26].

In the literature, there is a lack of consensus on the predictive value of specific parameters for sepsis in trauma patients, which depends on the studies reviewed. Additionally, evidence concerning how these various parameters behave in the context of the two age groups is scarce in the literature:

*Gender*: In our study, sex appeared to have little impact on sepsis development in either age group. Several studies identified male sex as an independent risk factor for sepsis in trauma patients [19,21,26], while other studies demonstrated that sex exerts no discernible influence in this regard [23,25].

*Trauma mechanism*: The trauma mechanism had no significant impact on the development of sepsis according to numerous studies [21,23,25,26], while an analysis by Kisat et al. revealed a significantly greater prevalence of penetrating injuries in patients with sepsis [19]. In contrast, our study revealed an association between blunt trauma and sepsis following polytrauma.

*ISS*: Patients with sepsis < 65 years had significantly greater ISSs than their non-septic counterparts. Many studies regarded the ISS as an independent risk factor for sepsis after severe trauma across all age groups [19,21,23,25,26]. A study by Fakhry et al. showed that preexisting comorbidities influenced the occurrence of sepsis more than the ISS did in geriatric polytrauma patients >65 years [27].

*GCS*: A lower GCS at the scene in patients aged <65 years and a higher GCS in patients aged ≥65 years was associated with an increased risk of sepsis. In a study by Chung et al., patients who developed sepsis following severe trauma had a significantly lower GCS at admission than patients without sepsis [26]. Likewise, the GCS at the scene was significantly lower for trauma patients with sepsis in a study by Wafaisade et al. [21]. Another study showed no significant differences in GCS levels at admission between trauma patients with and without sepsis [23]. Notably, none of the studies distinguished between age groups.

*Temperature*: Among patients with sepsis aged ≥65 years, the mean temperature was greater than that of patients without sepsis. Significantly greater temperatures in trauma patients with sepsis were also observed in a study by Tong et al.; however, the patients were not separated into different age groups [25].

*Leucocytes and CRP*: Upon admission, patients who developed sepsis tended to have significantly greater leucocyte counts in the age group < 65 years and higher CRP values in both age groups. A study by Park et al. revealed no statistically significant difference in leucocyte counts at admission between trauma patients with and without sepsis of all age groups [23]. Other studies demonstrated significantly higher leucocyte counts and CRP levels at admission in trauma patients with sepsis [25].

*pH and lactate*: In both age groups, acidosis and high lactate levels upon admission were not associated with sepsis. This was confirmed by a study by Tong et al., in which lactate levels at admission did not significantly differ between trauma patients with and without sepsis [25]. In contrast, according to a study conducted by Chung et al., elevated initial lactate levels were found to be an independent factor associated with sepsis following severe trauma across all age groups [26].

*Hemoglobin and hematocrit*: Lower hemoglobin and hematocrit values at admission were more meaningful indicators of sepsis in patients <65 years than in those aged ≥65 years. In a study by Park et al., age-independent hemoglobin levels at admission were not significantly different between trauma patients with and without sepsis [23]. Other studies revealed that trauma patients with sepsis exhibited significantly decreased hematocrit levels [25] or hemoglobin levels [21].

*Quick*: Patients with sepsis in both age groups did not show significant differences in Quick values at admission. Furthermore, the Quick did not appear to significantly affect the risk of sepsis. This statement is also supported by a study by Park et al. [23]. In contrast, Wafaisade et al. showed that Quick values at admission were significantly lower in trauma patients with sepsis than in patients without sepsis [21].

The development of sepsis, especially in geriatric polytrauma patients, often leads to death, with pneumonia being the most prevalent infection, followed by wound infections, urinary tract infections, and CRBSI. Therefore, it is of the utmost importance to detect and treat pneumonia early. Wounds should be treated according to established guidelines and regularly observed. Both urinary and blood catheters should be retained for as long as necessary but removed as quickly as possible.

Furthermore, the timing of surgical intervention plays a crucial role. Traditionally, the principle of “damage control surgery” (DCS) has been advocated, which involves a brief initial operation to control bleeding and prevent further tissue damage, followed by definitive surgery at a later stage when the patient’s physiological state has stabilized. This approach aims to minimize the risks associated with extensive surgery during a critical illness.

However, recent studies suggest that delaying definitive surgery for extended periods may be associated with an increased risk of sepsis [28,29]. The rationale behind this is that prolonged soft tissue exposure and the presence of foreign bodies, such as temporary implants used for provisional fixation, can create a nidus for infection. Additionally, the ongoing inflammatory response associated with trauma and tissue devitalization can further contribute to the development of sepsis. Delayed or prolonged immobilization can increase the likelihood of complications, including soft tissue damage, compartment syndrome, and impaired wound healing, all of which may predispose patients to infectious complications.

Therefore, striking a balance between achieving early source control and avoiding the complications of overly aggressive surgery during the acute phase is crucial. In the context of geriatric polytrauma patients, the decision making process becomes even more complex. These patients often have pre-existing comorbidities and a more fragile physiological reserve, making them potentially more susceptible to the complications associated with both early and delayed surgery.

## 5. Conclusions

Sepsis poses a formidable challenge in the context of geriatric polytrauma patients, where the limited parameters associated with sepsis make early detection and intervention particularly challenging. Evidence-based strategies to prevent sepsis include:

(1) Early antibiotics: broad-spectrum antibiotics within 1 h of suspected sepsis improve outcomes [30].
(2) Source control: address the infection source (surgery, drainage) to stop spread and inflammation [30].
(3) Monitoring: track vital signs, labs, and clinical indicators for early detection and intervention [30].

The destructive impact on the body necessitates a timely and vigorous response. Unfortunately, the road to septic recovery is prolonged, and the final outcome, especially in geriatric individuals, is often unfavorable. Recognizing the impending danger becomes crucial, and the principle of “HIT HARD and HIT EARLY” underscores the importance of proactive measures to counteract sepsis. The provided parameters must be considered together to decide whether preemptive therapy is meaningful or not, but one key question remains: at what point in time should preemptive therapy be initiated?

## Figures and Tables

**Table 1:** Descriptive statistics of the patient sample for patients <65 years and patients ≥65 years, including the number of total values.

Variable	*N*	Overall *N* = 3059 ^1^	Age Group		
			<65 Years *N* = 2416 ^1^	≥65 Years *N* = 643 ^1^	*p*-Value ^2^
Age [years]	3059	43 (28, 61)	37 (25, 49)	75 (69, 81)	<0.001
Male	3059	2250 (74%)	1867 (77%)	383 (60%)	<0.001
Blunt trauma	3059	2809 (92%)	2187 (91%)	622 (97%)	<0.001
ISS	3059	27 (22, 38)	28 (22, 38)	27 (22, 36)	0.9
AIS Head	3044	4.00 (1.00, 5.00)	4.00 (1.00, 5.00)	4.00 (3.00, 5.00)	<0.001
AIS Face	3008	0.00 (0.00, 1.00)	0.00 (0.00, 1.00)	0.00 (0.00, 0.00)	<0.001
AIS Thorax	3037	2.00 (0.00, 3.00)	2.00 (0.00, 3.00)	0.00 (0.00, 3.00)	<0.001
AIS Abdomen	3007	0.00 (0.00, 2.00)	0.00 (0.00, 3.00)	0.00 (0.00, 0.00)	<0.001
AIS Pelvis	3000	0.00 (0.00, 0.00)	0.00 (0.00, 0.00)	0.00 (0.00, 0.00)	0.5
AIS Extremities	3020	1.00 (0.00, 3.00)	2.00 (0.00, 3.00)	0.00 (0.00, 2.00)	<0.001
AIS Spine	3011	0.00 (0.00, 2.00)	0.00 (0.00, 2.00)	0.00 (0.00, 2.00)	0.018
AIS External	2988	0.00 (0.00, 1.00)	0.00 (0.00, 1.00)	0.00 (0.00, 1.00)	0.9
Length of ICU [days]	3038	5 (2, 12)	5 (2, 13)	2 (1, 7)	<0.001
Length of hospitalization [days]	3054	13 (4, 24)	15 (6, 25)	7 (2, 17)	<0.001
Death during hospitalization	3059	934 (31%)	611 (25%)	323 (50%)	<0.001
Death within 72 h	3051	683 (22%)	445 (18%)	238 (37%)	<0.001
Sepsis	3059	505 (17%)	430 (18%)	75 (12%)	<0.001
ATLS shock class	3022				0.002
1		1857 (61%)	1431 (60%)	426 (67%)	
2		736 (24%)	617 (26%)	119 (19%)	
3		218 (7.2%)	173 (7.2%)	45 (7.1%)	
4		211 (7.0%)	168 (7.0%)	43 (6.8%)	
GCS at site	2809	12.0 (4.0, 15.0)	12.0 (4.0, 15.0)	11.0 (5.0, 14.0)	0.6
Temperature at admission [°C]	2214	35.70 (34.70,36.58)	35.70 (34.70, 36.60)	35.70 (34.50, 36.40)	0.10
Leucocytes at admission [WBC/µL]	2795	12.3 (9.0, 16.4)	12.5 (9.2, 16.7)	11.5 (8.5, 14.9)	<0.001
CRP at admission [mg/L]	2349	3 (1, 5)	3 (1, 4)	3 (1, 7)	<0.001
pH at admission	2229	7.33 (7.26, 7.38)	7.33 (7.26, 7.38)	7.35 (7.27, 7.39)	0.011
Lactate at admission [mmol/L]	2591	2.30 (1.40, 3.60)	2.38 (1.50, 3.70)	2.00 (1.22, 3.10)	<0.001
Haemoglobin at admission [g/dL]	2607	11.70 (9.40, 13.30)	11.70 (9.40, 13.50)	11.40 (9.40, 12.90)	0.018
Haematocrit at admission [%]	2715	35 (28, 39)	35 (28, 40)	34 (28, 38)	0.065
Quick at admission [%]	2401	83 (62, 97)	84 (64, 97)	78 (57, 94)	<0.001
Damage Control Surgery	2883	1435 (50%)	1198 (53%)	237 (39%)	<0.001
Early Total Care	2883	973 (34%)	779 (34%)	194 (32%)	0.2
No intervention	2883	475 (16%)	293 (13%)	182 (30%)	<0.001

^1^ Median (IQR) or frequency (%). ^2^ Wilcoxon rank sum test; Pearson’s Chi-squared test.

**Table 2:** Welch’s *t*-tests displaying the central tendency of different parameters for non-sepsis and sepsis, separately for both age groups.

Variable	Age < 65 Years			Age ≥ 65 Years		
	Mean in Non-Sepsis Group	Mean in the Sepsis Group	*t*-Test *p*-Value	Mean in Non-Sepsis Group	Mean in the Sepsis Group	*t*-Test *p*-Value
ISS	30.76	33.85	t = −4.67 ***p* < 0.001**	32.58	33.99	t = −0.96 *p* = 0.34
Temperature at admission [°C]	35.53	35.37	t = 1.61 *p* = 0.11	35.26	35.74	t = −2.48 ***p* = 0.01**
Leucocytes at admission [WBC/µL]	13.23	13.94	t = −2.04 ***p* = 0.04**	12.07	13.09	t = −1.33 *p* = 0.19
CRP at admission [mg/L]	12.03	18.20	t = −2.07 ***p* = 0.04**	15.27	34.57	t = −1.94 *p* = 0.06
pH at admission	7.31	7.30	t = 0.84 *p* = 0.40	7.33	7.30	t = 1.66 *p* = 0.10
Lactate at admission [mmol/L]	3.16	2.96	t = 1.51 *p* = 0.13	2.63	2.78	t = −0.39 *p* = 0.69
Haemoglobin at admission [g/dL]	11.31	10.94	t = 2.31 ***p* = 0.02**	11.08	10.51	t = 1.57 *p* = 0.12
Haematocrit at admission [%]	33.60	32.40	t = 2.59 ***p* = 0.01**	33.05	31.26	t = 1.70 *p* = 0.09
Quick at admission [%]	78.70	77.21	t = 1.23 *p* = 0.22	72.25	73.52	t = −0.09 *p* = 0.93

Bold numbers indicate significant *p*-values.

**Table 3:** Univariate logistic regression analysis of factors for sepsis stratified by age (odds ratios are presented along with 95% confidence intervals).

Variable	Sepsis in Patients < 65 y			Sepsis in Patients ≥ 65 y		
	OR ^1^	95% CI ^1^	*p*-Value	OR ^1^	95% CI ^1^	*p*-Value
Male	1.22	0.94, 1.58	0.14	1.41	0.86, 2.38	0.2
Blunt trauma	1.69	1.13, 2.62	**0.013**	2.70	0.55, 48.8	0.3
ISS	1.02	1.01, 1.02	**<0.001**	1.00	0.99, 1.02	0.5
AIS Head	1.07	1.01, 1.13	**0.014**	0.80	0.71, 0.90	**<0.001**
AIS Face	1.00	0.91, 1.10	>0.9	1.07	0.84, 1.35	0.6
AIS Thorax	1.13	1.06, 1.20	**<0.001**	1.33	1.16, 1.53	**<0.001**
AIS Abdomen	1.07	1.01, 1.13	**0.020**	1.37	1.18, 1.59	**<0.001**
AIS Pelvis	1.11	1.03, 1.20	**0.008**	1.37	1.16, 1.61	**<0.001**
AIS Extremities	1.09	1.01, 1.16	**0.022**	1.43	1.23, 1.68	**<0.001**
AIS Spine	1.04	0.97, 1.11	0.3	1.31	1.12, 1.51	**<0.001**
AIS External	0.99	0.87, 1.12	>0.9	1.28	0.96, 1.68	0.082
ATLS shock class	1.15	1.02, 1.28	**0.016**	1.20	0.93, 1.53	0.15
GCS at site	0.98	0.96, 1.00	0.072	1.07	1.01, 1.13	**0.019**
Temperature at admission	0.95	0.89, 1.01	0.12	1.21	1.01, 1.48	0.056
Leucocytes at admission	1.02	1.00, 1.04	**0.027**	1.03	0.99, 1.08	0.14
CRP at admission	1.00	1.00, 1.01	**0.010**	1.01	1.00, 1.01	**0.007**
pH at admission	0.69	0.31, 1.61	0.4	0.15	0.02, 1.52	0.094
Lactate at admission	0.97	0.93, 1.01	0.2	1.03	0.91, 1.13	0.6
Haemoglobin at admission	0.96	0.93, 1.00	**0.027**	0.93	0.85, 1.02	0.11
Haematocrit at admission	0.98	0.97, 1.00	**0.012**	0.97	0.94, 1.00	0.083
Quick at admission	1.00	0.99, 1.00	0.2	1.00	0.99, 1.01	>0.9

^1^ OR = odds ratio, CI = confidence interval. Bold numbers indicate significant *p*-values.

**Table 4:** Multivariate logistic regression analysis of identified risk factors for sepsis stratified by age (odds ratios are presented along with 95% confidence intervals).

Variable	Sepsis in Patients < 65 y			Sepsis in Patients ≥ 65 y		
	OR ^1^	95% CI ^1^	*p*-Value	OR ^1^	95% CI ^1^	*p*-Value
Blunt trauma	1.85	1.08, 3.38	**0.034**	4,302,671	0.00, NA	>0.9
ISS	1.02	1.01, 1.03	**<0.001**	1.01	0.99, 1.03	0.5
ATLS shock class	1.11	0.94, 1.31	0.2	1.15	0.81, 1.61	0.4
GCS at site	0.97	0.95, 1.00	0.055	1.11	1.03, 1.20	**0.006**
Leucocytes at admission	1.04	1.02, 1.06	**<0.001**	1.03	0.98, 1.08	0.2
CRP at admission	1.00	1.00, 1.01	**0.048**	1.01	1.00, 1.01	**<0.001**
Haemoglobin at admission	1.22	0.99, 1.53	0.076	1.42	0.82, 2.43	0.2
Haematocrit at admission	0.93	0.86, 1.00	0.053	0.86	0.72, 1.04	0.11

^1^ OR = odds ratio, CI = confidence interval, NA = not available. Bold numbers indicate significant *p*-values.

**Table 5:** Source of infection in polytrauma patients who developed sepsis.

Source of Infection	Overall *N* = 505 ^1^	Age Group		
		<65 Years *N* = 430 ^1^	≥65 Years *N* = 75 ^1^	*p*-Value ^2^
Pneumonia	347 (69%)	291 (68%)	56 (75%)	0.2
Bacteraemia	182 (36%)	154 (36%)	28 (37%)	0.8
CRBSI	125 (25%)	111 (26%)	14 (19%)	0.2
Wound infection	102 (20%)	84 (20%)	18 (24%)	0.4
UTI	80 (16%)	65 (15%)	15 (20%)	0.3
CNS infection	44 (8.7%)	42 (9.8%)	2 (2.7%)	**0.044**
Other infection	44 (8.7%)	36 (8.4%)	8 (11%)	0.5
Intraabdominal infection	32 (6.3%)	28 (6.5%)	4 (5.3%)	>0.9
Osteomyelitis	9 (1.8%)	9 (2.1%)	0 (0%)	0.4

^1^ Frequency (%). ^2^ Pearson’s Chi-squared test. Bold numbers indicate significant *p*-values.

## Data Availability

All the data are available upon reasonable request. None of these data are available to a broad public. All the data are stored in the clinical information system (KISIM) of the University Hospital Zurich.
